# LocoMMotion: a study of real-life current standards of care in triple-class exposed patients with relapsed/refractory multiple myeloma – 2-year follow-up (final analysis)

**DOI:** 10.1038/s41375-024-02404-6

**Published:** 2024-09-25

**Authors:** María-Victoria Mateos, Katja Weisel, Valerio De Stefano, Hartmut Goldschmidt, Michel Delforge, Mohamad Mohty, Dominik Dytfeld, Emanuele Angelucci, Laure Vincent, Aurore Perrot, Reuben Benjamin, Niels W. C. J. van de Donk, Enrique M. Ocio, Tito Roccia, Jordan M. Schecter, Silva Koskinen, Imène Haddad, Vadim Strulev, Lada Mitchell, Jozefien Buyze, Octavio Costa Filho, Hermann Einsele, Philippe Moreau

**Affiliations:** 1 Hospital Universitario de Salamanca, Instituto de Investigación Biomédica de Salamanca (IBASL), Centro de Investigación del Cáncer (IBMCC-USAL,CSIC), Salamanca, Spain; 2 University Medical Center Hamburg-Eppendorf, Hamburg, Germany; 3 Catholic University, Fondazione Policlinico A. Gemelli, IRCCS, Rome, Italy; 4 University Hospital Heidelberg, Heidelberg, Germany; 5 University of Leuven, Leuven, Belgium; 6 Sorbonne University, Saint-Antoine Hospital, AP-HP INSERM UMRs 938, Paris, France; 7 Poznan University of Medical Sciences, Poznań, Poland; 8 Hematology and Cellular Therapy, IRCCS Ospedale Policlinico San Martino, Genova, Italy; 9 Centre Hospitalier Universitaire de Montpellier, Montpellier, France; 10 Centre Hospitalier Universitaire de Toulouse, Service d’Hématologie, Toulouse, France; 11 School of Cancer and Pharmaceutical Sciences, King’s College London, London, UK; 12 Amsterdam University Medical Center, Vrije Universiteit Amsterdam, Amsterdam, Netherlands; 13 Hospital Universitario Marqués de Valdecilla (IDIVAL) Universidad de Cantabria, Santander, Spain; 14 Janssen Global Services, Raritan, NJ USA; 15 Janssen Research & Development, Raritan, NJ USA; 16 Janssen-Cilag, Espoo, Finland; 17 Janssen-Cilag, Issy-les-Moulineaux, France; 18 Janssen Pharmaceutica NV, Beerse, Belgium; 19 J&J, Allschwil, Switzerland; 20 Legend Biotech USA Inc, Somerset, NJ USA; 21 Universitätsklinikum Würzburg, Medizinische Klinik und Poliklinik II, Würzburg, Germany; 22 University Hospital Hotel-Dieu, Nantes, France

**Keywords:** Clinical trials, Cancer therapy

## Abstract

Treatment of relapsed/refractory multiple myeloma (RRMM) is challenging as patients exhaust all available therapies and the disease becomes refractory to standard drug classes. Here we report the final results of LocoMMotion, the first prospective study of real-world clinical practice (RWCP) in triple-class exposed (TCE) patients with RRMM, with a median follow-up of 26.4 months (range, 0.1–35.0). Patients (*N*  =  248) had received median 4 prior LOT (range, 2–13) at enrollment. 91 unique regimens were used in index LOT. Overall response rate was 31.9% (95% CI, 26.1–38.0), median progression-free survival (PFS) was 4.6 months (95% CI, 3.9–5.6) and median overall survival was 13.8 months (95% CI, 10.8–17.0). 152 patients (61.3%) had subsequent LOTs with 134 unique regimens, of which 78 were used in first subsequent LOT. Median PFS2 (from start of study through first subsequent LOT) was 10.8 months (95% CI, 8.4–13.0). 158 patients died on study, 67.7% due to progressive disease. Additional subgroup analyses and long-term safety summaries are reported. The high number of RWCP treatment regimens utilized and poor clinical outcomes confirm a lack of standardized treatment for TCE patients with RRMM, highlighting the need for new treatments with novel mechanisms.

## Introduction

With recent advances in multiple myeloma (MM) treatment, patients with MM are living longer [1–3]. However, because most patients relapse and/or their disease becomes refractory to treatment [4, 5], they cycle through standard drug classes, including proteasome inhibitors (PIs), immunomodulatory drugs (IMiDs), anti-CD38 monoclonal antibodies, and others. Despite a wide array of conventional treatment options for relapse/refractory MM (RRMM), periods of remission are generally short in real-world clinical practice (RWCP) [6] and outcomes worsen with each subsequent line of therapy (LOT) [5, 6], making treatment selection progressively more challenging [4, 5, 7].

LocoMMotion (ClinicalTrials.gov identifier: NCT04035226) was the first multinational, prospective, observational study to examine effectiveness and safety of RWCP therapies in triple-class exposed patients with MM. Its prospective study design allowed for collection of patient-level baseline characteristics and outcome parameters that are not commonly collected during routine clinical practice. Previous results from LocoMMotion, reported at 16.1 months median study follow-up, showed a lack of clear standard of care for treatment of triple-class exposed patients and poor outcomes [8]. Ninety-one unique regimens were used in the index LOT (first treatment after enrollment), and the overall response rate (ORR) was 31.5%, with median progression-free survival (PFS) and overall survival (OS) of 4.6 months and 13.8 months, respectively. Treatment-emergent adverse events (TEAEs) were reported in 85.9% of patients, with 56.5% reporting grade 3/4 TEAEs. Here, we report results from the final analysis of LocoMMotion, including mature survival data, additional subgroup analyses, subsequent therapies, and their outcomes, as well as long term safety data, including second primary malignancies (SPM), and TEAEs.

## Patients and methods

### Study design and treatment

LocoMMotion was a prospective, observational study that explored the use of RWCP therapies in the management of triple-class exposed patients with RRMM (i.e., had received a PI, an IMiD, and an anti-CD38 monoclonal antibody [mAb]). Eligible patients had received ≥3 prior LOT or their disease was double refractory to a PI and an IMiD, were triple-class exposed, and had documented disease progression during or after their last LOT. Further eligibility criteria included age ≥18 years with a documented diagnosis of MM per International Myeloma Working Group (IMWG) criteria [9–11], measurable disease assessed by serum free light chain (≥10 mg/dL and abnormal ratio) or M-protein (≥1.0 g/dL [serum] or ≥200 mg/24 h [urine]), and an Eastern Cooperative Oncology Group performance status (ECOG PS) of 0 or 1. Patients were enrolled across 75 sites in Europe (Belgium, France, Germany, Italy, Netherlands, Poland, Russia, Spain, and the United Kingdom) and the United States.

LocoMMotion included 3 phases: 1) a screening phase (the 28-day period prior to enrollment) in which baseline patient and disease characteristics, diagnosis, and medication history were collected; 2) an index LOT phase (time from the first day of on-study RWCP treatment until initiation of subsequent antimyeloma therapy) in which treatment type, effectiveness data, and safety data were collected; and 3) a follow-up phase until study completion wherein patients were followed for documentation of subsequent LOT, PFS2, and OS. Patients could receive ≥1 regimen during a LOT (e.g., if they started with triplet therapy and were de-escalated to a doublet therapy due to toxicity). Patients were allowed to enroll in other trials and/or use experimental therapies in subsequent LOT. End of study was defined as 24 months after the first dosing of the last patient enrolled. RWCP treatments were defined as those approved and used in local clinical practice for treatment of adult patients with RRMM. During index LOT, a response review committee (RRC) comprising three MM hematologists reassessed responses and evaluated disease progression in accordance with IMWG criteria, in a blinded manner, to ensure consistency of the assessments. Responses with subsequent LOT were subject to investigator assessment.

This study was conducted in accordance with the Declaration of Helsinki. Written informed consent was provided by all patients, and the study protocol was approved by an independent ethics committee/institutional review board at each center (Supplementary Table S1).

### Endpoints and assessments

The primary effectiveness endpoint of the LocoMMotion study was ORR during treatment with index LOT, as assessed by the RRC. ORR was defined as the proportion of patients achieving partial response or better according to the IMWG criteria. Secondary effectiveness endpoints included rates of stringent complete response, complete response (CR), very good partial response (VGPR), duration of response (DOR), PFS, time to first response, and time to best response. These were assessed by RRC during treatment with index LOT. Investigator assessments were also collected for secondary effectiveness endpoints: time to next treatment, ORR, PFS, PFS2, and OS.

DOR was defined as time from first documentation of partial response or better to first documented evidence of progressive disease (PD) as assessed by RRC or death, whichever occurred first, or start of subsequent therapy. PFS was defined as time from day 1 of index LOT to first documented evidence of PD by RRC, per IMWG response criteria, or death by any cause, whichever occurred first; data were censored at the last disease evaluation before the start of subsequent therapy. PFS2 was defined as the time between day 1 of index LOT and either the first date of documented PD by the study investigator after the start of the first subsequent LOT, or death from any cause from the start of the study. PFS2 included death events from the start of index LOT and PD events from the start of the first subsequent LOT (i.e., not PD events during index LOT). For patients who did not progress on index LOT and who were alive, data were censored prior to the start of the first subsequent LOT. Patients who withdrew before the first subsequent LOT were also censored. Time to next treatment was defined as the time from the first day of index LOT to initiation of subsequent antimyeloma therapy or death. For participants who did not receive subsequent antimyeloma therapy and are alive, data were censored at the last disease evaluation. OS was measured from day 1 of index LOT to the date of the patient’s death, with censoring for patients who were alive at study completion. Prespecified subgroup analysis was performed across a range of outcomes, including PFS, OS (reporting number of events, median time in months, and corresponding 95% confidence intervals [CIs]), and ORR.

Safety assessments included incidence and severity of adverse events (AEs), including TEAEs and second primary malignancies. TEAEs included all AEs that occurred between the start of and 30 days after the end of index LOT or the day prior to the first subsequent LOT, whichever occurred first. TEAEs also included AEs considered related to the study drug, regardless of start date, and AEs present at baseline that worsened.

### Statistical analyses

As LocoMMotion was an observational study, no direct hypothesis was tested, and the sample size was based on the clinically acceptable precision of the 95% CI for the primary objective, as previously described [12]. For the primary endpoint, exact 95% CIs were calculated by the Clopper-Pearson method. Variables were summarized using descriptive statistics. Time-to-event data were summarized using the Kaplan–Meier method. Categorical values were summarized using the number of observations and percentages. Subgroup analysis was reported as descriptive summaries and graphically presented using forest plots. Additional measures applied to account for potential missing assessments in RWCP have been previously described [12].

## Results

### Patients

**Fig. 1:**
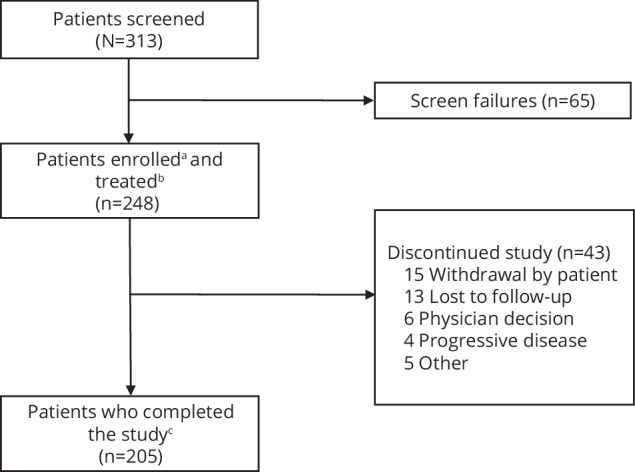
Study disposition. ^a^Enrolled patients are those who signed informed consent and were formally enrolled into the study. ^b^Treated patients are those who were enrolled into the study and received at least 1 real-world clinical practice treatment. ^c^Patients who completed the study are those who either died or remained on study at the end of the study (24 months), whichever occurred first.

Of 313 patients screened for the LocoMMotion study, 248 patients were enrolled between August 2, 2019, and October 26, 2020 (all-treated analysis set). As previously reported, 225 (90.7%) patients were from Europe and 23 (9.3%) were from the United States. At the end of study in October 2022, median study follow-up was 26.4 months (range, 0.1–35.0), and 205 (82.7%) patients had completed the study, including 158 (63.7%) patients who died. Forty-three (17.3%) patients had discontinued, including 13 who were lost to follow-up (Fig. 1). Patient and disease baseline characteristics have been previously described [12]. Median age was 68 years (range, 41–89), 87 (40.1%) had creatinine clearance ≤60 mL/min, 81 (40.5%) had lactate dehydrogenase levels >245 U/L, and 135 patients (54.4%) were male. At baseline, patients had received a median of 4 prior LOT (range, 2–13), 25.0% had received 4 prior LOT, and 49.2% had received ≥5 prior LOT. One hundred eighty-two (73.4%) patients were triple-class refractory, 229 (92.3%) were refractory to the last LOT, and 160 (64.5%) had prior stem cell transplantation. Median time to triple-class exposure was 4.7 years (range, 0–20.4).

### Index LOT

Ninety-one unique RWCP treatment regimens were used by patients in the index LOT, including a range of combinations of glucocorticoids (91.5%), PIs (54.4%), IMiDs (48.8%), and anti-CD38 monoclonal antibodies (9.7%) (Table 1). Six (2.4%) patients received stem cell transplantation (all autologous), and 7 patients (2.8%) received BCMA-targeted therapy. In total, 162 (65.3%) patients received a combination of ≥3 drugs. The most commonly received index regimens were carfilzomib-dexamethasone (Kd, 14.1%), pomalidomide-cyclophosphamide-dexamethasone (PCd, 14.1%), and pomalidomide-dexamethasone (Pd, 11.7%); most other regimens were used by fewer than 5 patients (15 regimens were used by only 2 patients and 46 were used by only 1 patient each). Patients received a median of 4 (range, 1–37) cycles of index treatment; median duration of treatment was 4.0 months (range, 0.1–33.6). Two hundred twenty-one patients (89.1%) discontinued index treatment, most commonly due to PD (54.8%), death (8.5%), and physician decision (8.5%). Twenty-seven patients completed the study.

**Table 1:** Antimyeloma RWCP treatments during index and subsequent LOT.

Treatment, *n* (%)^a^	Index LOT (*N* = 248)	1st Subsequent LOT (*n* = 152)	2nd Subsequent LOT (*n* = 74)	3rd Subsequent LOT (*n* = 42)	≥4th Subsequent LOT (*n* = 17)
Glucocorticoids	227 (91.5)	107 (70.4)	47 (63.5)	23 (54.8)	9 (52.9)
PI	135 (54.4)	53 (34.9)	24 (32.4)	9 (21.4)	8 (47.1)
Carfilzomib	63 (25.4)	22 (14.5)	11 (14.9)	3 (7.1)	3 (17.6)
Bortezomib	50 (20.2)	24 (15.8)	10 (13.5)	5 (11.9)	6 (35.3)
Ixazomib	22 (8.9)	7 (4.6)	3 (4.1)	1 (2.4)	1 (5.9)
IMiD	121 (48.8)	51 (33.6)	17 (23)	10 (23.8)	7 (41.2)
Pomalidomide	74 (29.8)	30 (19.7)	7 (9.5)	6 (14.3)	4 (23.5)
Lenalidomide	36 (14.5)	9 (5.9)	5 (6.8)	3 (7.1)	5 (29.4)
Thalidomide	11 (4.4)	11 (7.2)	5 (6.8)	1 (2.4)	1 (5.9)
Iberdomide	0 (0)	1 (0.7)	0 (0)	0 (0)	0 (0)
Alkylating agents	108 (43.5)	68 (44.7)	29 (39.2)	13 (31)	5 (29.4)
Cyclophosphamide	80 (32.3)	43 (28.3)	17 (23)	7 (16.7)	4 (23.5)
Bendamustine	16 (6.5)	14 (9.2)	6 (8.1)	2 (4.8)	0 (0)
Melphalan	14 (5.6)	13 (8.6)	6 (8.1)	4 (9.5)	0 (0)
Carmustine	1 (0.4)	1 (0.7)	1 (1.4)	1 (2.4)	0 (0)
Other	1 (0.4)	0 (0)	0 (0)	1 (2.4)	0 (0)
Melflufen	0 (0)	0 (0)	1 (1.4)	0 (0)	1 (5.9)
Anti-CD38 mAb	24 (9.7)	16 (10.5)	5 (6.8)	11 (26.2)	7 (41.2)
Daratumumab	23 (9.3)	13 (8.6)	4 (5.4)	5 (11.9)	6 (35.3)
Isatuximab	1 (0.4)	3 (2)	1 (1.4)	6 (14.3)	2 (11.8)
BCMA-targeted therapies	7 (2.8)	24 (15.8)	21 (28.4)	10 (23.8)	7 (41.2)
CAR-T	0 (0)	0 (0)	2 (2.7)	0 (0)	1 (5.9)
Bispecific antibody	0 (0)	5 (3.3)	2 (2.7)	0 (0)	4 (23.5)
Antibody-drug conjugate	7 (2.8)	19 (12.5)	17 (23)	10 (23.8)	3 (17.6)
Anthracyclines	21 (8.5)	8 (5.3)	7 (9.5)	4 (9.5)	2 (11.8)
Topoisomerase inhibitor	17 (6.9)	6 (3.9)	3 (4.1)	1 (2.4)	0 (0)
Histone deacetylase inhibitor	12 (4.8)	6 (3.9)	0 (0)	0 (0)	0 (0)
Anti-SLAMF7 mAb	9 (3.6)	7 (4.6)	0 (0)	1 (2.4)	2 (11.8)
BCL-2 inhibitor	6 (2.4)	3 (2)	2 (2.7)	2 (4.8)	1 (5.9)
Selective inhibitor of nuclear export	2 (0.8)	11 (7.2)	3 (4.1)	3 (7.1)	4 (23.5)
Other	19 (7.67)	9 (5.9)	7 (9.5)	4 (9.5)	4 (23.5)

*BCMA* B-cell maturation antigen, *Bcl* B-cell lymphoma, *CAR* chimeric antigen receptor, *IMiD* immunomodulatory drug, *LOT* line of therapy, *mAb* monoclonal antibody, *RWCP* real-world clinical practice, *PI* proteasome inhibitor, *SLAM* signaling lymphocytic activation molecule.

^a^Patients can be counted in >1 drug group.

### Subsequent LOT

**Fig. 2:**
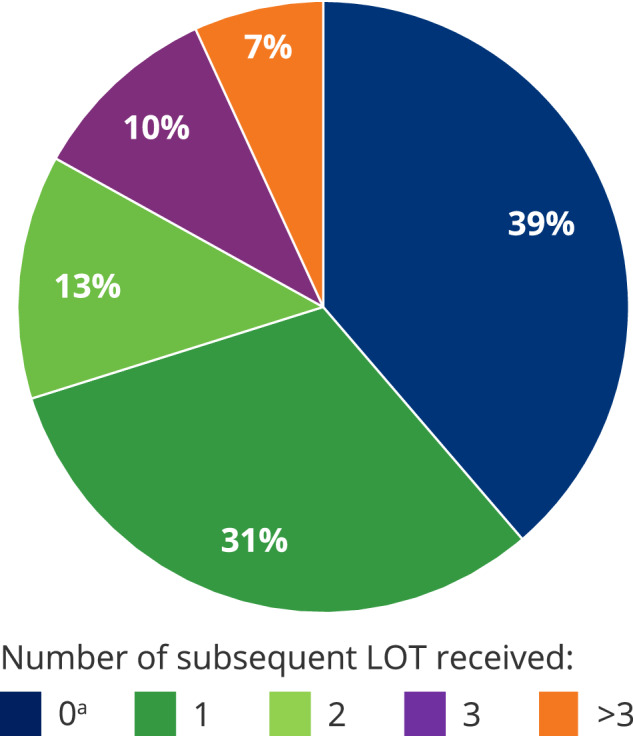
Patients receiving subsequent LOT. *LOT* line of therapy. ^a^Includes 36 (14.5%) patients who remained on index LOT.

One hundred fifty-two patients (61.3%) received at least 1 subsequent LOT after the index LOT (Supplementary Table S2). One hundred thirty-four unique regimens were used; 19 regimens were used by only 2 patients and 89 by only 1 patient. Overall, the most commonly used first subsequent LOTs were belantamab mafodotin (10.5% of those who received subsequent LOT) and PCd (6.6%; Table 2). A range of doublet and triplet therapies were also used during subsequent LOT, including daratumumab-carfilzomib-dexamethasone, isatuximab-carfilzomib-dexamethasone, Kd, Pd, and isatuximab-pomalidomide-dexamethasone. Compared to index LOT, more patients received BCMA-targeted therapy as subsequent LOT (2.8% during index vs 39.5% of those who received subsequent LOT). Overall, patients spent a median 4.5 months (range, 0.03–29.70) on subsequent LOT. Seventy-eight patients (31.5% of enrolled patients) had only 1 subsequent LOT, and 74 (29.8%) had ≥2 subsequent LOT (Fig. 2).

**Table 2:** Most common regimens used across index and subsequent LOT.

LOT	Number of unique regimens	Most common regimens,^a^ (%)
Index LOT	91	Kd (14.1), CPd (14.1), Pd (11.7)
1st Subsequent LOT (*n* = 152)	78	blmf (10.5), PCd (6.6)
2nd Subsequent LOT (*n* = 74)	47	blmf (18.9), KCd (5.4), Kd (5.4)
3rd Subsequent LOT (*n* = 42)	30	blmf (19.0), IsaPd (11.9), DKd (4.8)
≥4th Subsequent LOT (*n* = 17)	34	blmf (17.6)

*blmf* belantamab mafodotin, *PCd* pomalidomide, cyclophosphamide, and dexamethasone, *DKd* daratumumab, carfilzomib, and dexamethasone, *IsaPd* isatuximab, pomalidomide, and dexamethasone, *KCd* carfilzomib, cyclophosphamide, and dexamethasone, *Kd* carfilzomib and dexamethasone, *LOT* line of therapy, *Pd* pomalidomide and dexamethasone.

^a^Patients can be counted in >1 regimen per LOT.

### Effectiveness: Index LOT

ORR with index treatment, assessed by RRC, for patients treated with RWCP therapies was 31.9% (95% CI, 26.1–38.0), median duration of treatment was 4.0 months (range, 0.1–33.6), and median DOR was 7.4 months (95% CI, 4.9–11.1). Overall best response for patients was generally unchanged from the previous data cut at 16.1 months median study follow-up [8]. No patients achieved stringent complete response, 1 patient (0.4%) achieved CR, 32 (12.9%) achieved VGPR, 46 (18.5%) achieved PR, 14 (5.6%) had minimal response, 78 (31.5%) had stable disease, and 43 (17.3%) had PD. Thirty-four patients (13.7%) were not evaluable by RRC (mainly due to death, AEs, or rapid disease progression requiring a switch to another treatment, occurring before confirmation of response). Among the 34 RRC-unevaluable patients, investigators assessed 3 as responders and 31 as not evaluable, PD, stable disease, or minimal response.

**Fig. 3:**
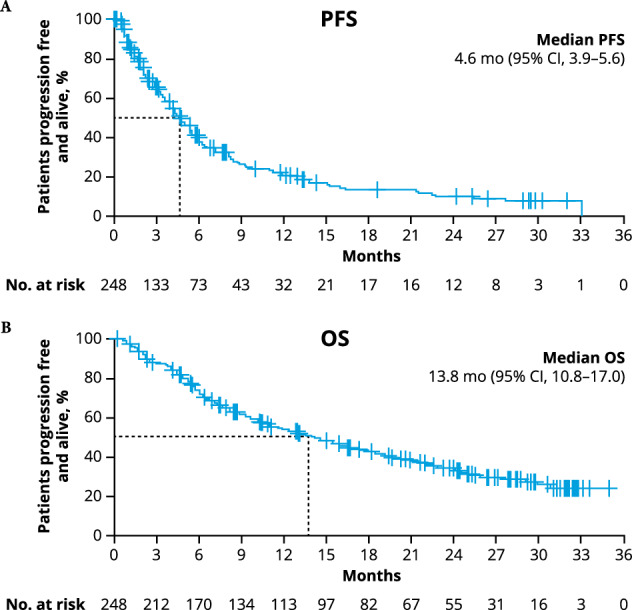
Survival outcomes with RWCP therapies. Kaplan–Meier plots showing (**A**) progression-free survival and **B** overall survival based on RRC assessment at median study follow-up 26.4 months in all patients. OS overall survival, PFS progression-free survival, RRC response review committee, VGPR very good partial response.

In RRC-evaluable responders (*n* = 79), median time to first response was 1.9 months (range, 0.7–25.8), and median time to best response was 2.4 months (range, 0.7–25.8). ORR results as assessed by investigators (34.7% [95% CI, 28.8–41.0%]) were similar (85.9% concordant). Median PFS by RRC was 4.6 months and median OS was 13.8 months (Fig. 3). Twelve- and 24-month PFS rates were 21.0% (95% CI, 15.3–27.3) and 10.5% (95% CI, 6.1–16.3); 12- and 24-month OS rates were 53.4% (95% CI, 46.7–59.6) and 33.7% (95% CI, 27.3–40.2), respectively.

**Fig. 4:**
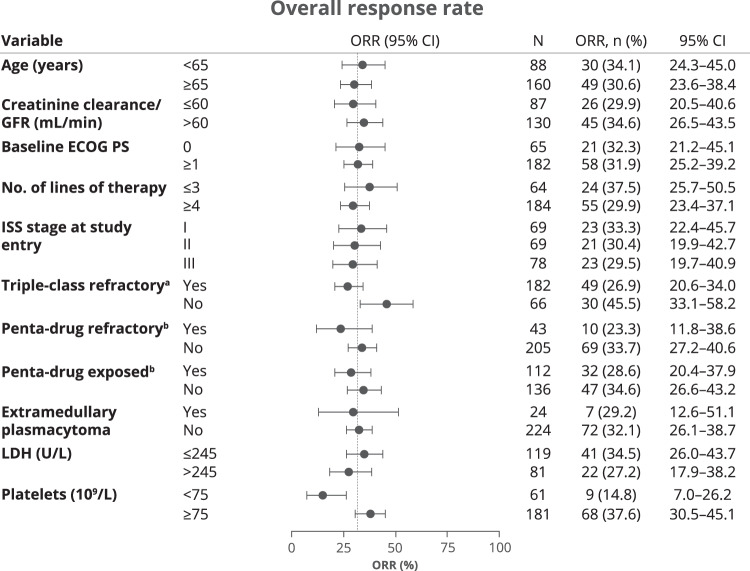
Forest plot of subgroup analyses of overall response rate by RRC. ECOG Eastern Cooperative Oncology Group, GFR glomerular filtration rate, ISS International Staging System, LDH lactate dehydrogenase, PS performance status, RRC response review committee. ^a^Triple-class exposed/refractory is defined as exposed/refractory to a proteasome inhibitor (PI), an immunomodulatory drug (IMiD), and an anti-CD38 antibody. ^b^Penta-drug exposed/refractory is defined as exposed/refractory to at least 2 PIs, 2 IMiDs, and 1 anti-CD38 antibody (includes triple-class exposed/refractory).

Prespecified subgroup analysis showed that ORR was minimally impacted by high-risk disease characteristics at baseline, including ≥4 prior LOT (29.9%), International Staging System (ISS) stage III at study entry (29.5%), extramedullary plasmacytomas (29.2%), penta-drug refractory MM (23.3%), and penta-drug exposed MM (28.6%) (Fig. 4). ORRs were lower in patients who had a low level of thrombocytes (<75 × 10^9^/L) at baseline (14.8% vs 37.6% in those with thrombocyte levels ≥75 × 10^9^/L) and those who had triple-class refractory MM (26.9% vs 45.5% in patients who did not have triple-class refractory MM) than in patients without these risk factors. Missing data prevented interpretation of data for subgroups by cytogenetic risk and percentage of bone marrow plasma cells. Several risk factors also impacted PFS outcomes, including extramedullary plasmacytomas (median 2.7 months with plasmacytomas vs 5.1 months without plasmacytomas), triple-class refractoriness (4.1 vs 8.2 months in patients who did not have triple-class refractory MM), penta-refractoriness (3.4 vs 5.5 months in patients who did not have penta-drug refractory MM), low thrombocyte levels (3.1 vs 5.6 months in patients with ≥75 × 10^9^/L thrombocytes), and lactate dehydrogenase (LDH) > 245 U/L (3.4 vs 5.6 months in patients with LDH ≤ 245 U/L) (Supplementary Fig. S1). OS outcomes were worse in patients who had baseline ECOG PS ≥ 1 (median 11.1 months vs 24.1 months in those with ECOG PS 0), ISS stages II or III (stage II: 9.7 months and stage III: 12.4 months vs stage I: 24.0 months), LDH > 245 U/L (7.4 vs 17.0 months in patients with LDH ≤ 245 U/L), low thrombocyte levels (5.8 vs 18.1 months in patients with ≥75 × 10^9^/L thrombocytes), and those who had penta-drug refractory MM (8.2 vs 15.3 months in patients who did not have penta-drug refractory MM).

Outcomes were worse for patients who did not (*n* = 215) versus those who did achieve (*n* = 33) ≥ VGPR. Median PFS was 3.9 months in patients who did not achieve ≥VGPR versus 15.2 months in patients who did achieve ≥VGPR, and median OS was 10.9 months versus not estimable. Patients who achieved ≥VGPR showed significantly longer median DOR versus those who achieved PR (13.1 months vs 4.7 months).

### Effectiveness: subsequent LOT

Estimated median time from start of index LOT to next treatment was 5.2 months (95% CI, 4.4–6.0). Median duration of treatment with first subsequent LOT was 2.8 months (range, <1–29.7). Median PFS2 by investigator assessment was 10.8 months (95% CI, 8.4–13.0).

### Safety

During index LOT, 86.7% of patients experienced an AE; grade 3/4 TEAEs occurred in 144 (58.1%) patients (Table 3). The most common TEAEs were hematologic, occurring in 50% of patients, and included thrombocytopenia (any grade 26.2%, grade 3/4 19.4%), anemia (any grade 25.8%, grade 3/4 10.9%), and neutropenia (any grade 20.2%, grade 3/4 17.3%) (Supplementary Table S3). The most common non-hematologic TEAEs (all grades; reported in ≥15% patients) were general disorders and administration site conditions (40.7%), gastrointestinal disorders (33.5%), and infections and infestations (33.1%). No grade 3/4 non-hematological TEAEs occurred in ≥15% of patients. SPMs were reported in 13 (5.2%) patients; 5 were reported during index LOT and 8 were reported after index LOT. Cases of SPMs included squamous cell carcinoma (*n* = 4), basal cell carcinoma (*n* = 2), secondary acute myeloid leukemia (*n* = 2), cancer of the lung/bronchus (*n* = 2), and 1 case each of high grade suspected cholangio cellular carcinoma, plasma cell leukemia, and an SPM described as multiple destructive fluorodeoxyglucose (FDG)-positive lesions throughout the skeleton and skull. Of note, the case of plasma cell leukemia and of SPM with multiple destructive FDG-positive lesions may have been MM; however, they were reported as SPM by the investigators. Only 1 SPM case (plasma cell leukemia) was reported as fatal.

**Table 3:** Severity of TEAEs during index LOT.

TEAE, *n* (%)	*N* = 248
Any TEAE	215 (86.7)
Any serious TEAE	91 (36.7)
Maximum severity of TEAE	
Grade 1	17 (6.9)
Grade 2	46 (18.5)
Grade 3	84 (33.9)
Grade 4	46 (18.5)
Grade 5	22 (8.9)
TEAE with outcome death	21 (8.5)^a^

*LOT* line of therapy, *PD* progressive disease, *TEAE* treatment-emergent adverse event.

^a^1 patient with unresolved grade 5 TEAE died from PD.

**Fig. 5:**
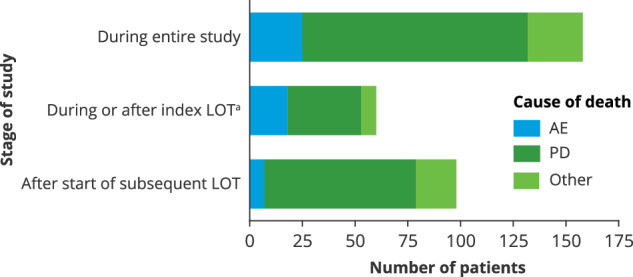
Deaths and causes of death. AE adverse event, PD progressive disease, LOT line of therapy. ^a^These patients did not receive subsequent LOT.

Overall, 158 (63.7%) patients died during the study. Most deaths were due to PD (67.7%), 15.8% were due to AEs, and 16.5% were due to other causes (Fig. 5). Sixty deaths occurred during or after index LOT (but before subsequent LOT) and 98 occurred after the start of subsequent LOT. During index LOT, 21 (8.5%) patients died from TEAEs, most commonly infection (13 patients, 5.2%), and 1 patient had a grade 5 TEAE that did not resolve before the patient died from PD.

## Discussion

LocoMMotion was the first prospective, observational study to explore the effect of RWCP treatments in triple-class exposed patients with RRMM. The high number of unique regimens in the index and subsequent LOTs, including that more than two-thirds of unique regimens were used by fewer than 5 patients, demonstrates a lack of established standard-of-care therapy in this population. Triple-class exposed patients showed rapid disease progression on index LOT and with first subsequent LOT and poor survival despite the use of a wide range of treatments during the study. Response rate to index LOT was low (79 patients, 31.9%), and responses were neither deep nor durable. OS, PFS, and DOR were longer in patients who achieved ≥VGPR; however, only 1 in 10 were able to achieve this level of response with the observed treatments. Median PFS and median OS were short at 4.6 months and 13.8 months, respectively. Similar to other studies [13, 14], OS outcomes were worse in patients who had higher baseline ECOG PS, ISS stage II or III, high LDH, penta-drug refractory disease, and low levels of thrombocytes. ORR was less sensitive to these factors, as it was impacted only by low levels of thrombocytes and triple-refractoriness. However, greater depth of response did correspond to improved PFS and OS outcomes, suggesting that achievement of ≥VGPR is an important treatment goal in RWCP.

Across first subsequent LOTs, median PFS2 by investigator assessment was 10.8 months, which includes a median of 4.6 months for index LOT. Thus, PFS after subsequent LOT was similar to that after index LOT. This highlights the continuous short cycles of remission and subsequent relapses, and the increasing challenge that patients and clinicians face in determining the next treatment choice.

Limitations include the study enrollment period, which occurred between 2019 and 2020, and does not represent novel therapies approved since 2020, including chimeric antigen receptor T-cell therapies and bispecific antibodies. While the United States population (9.3%) was represented in this study, generalizability of the study to the United States might be limited by frontline treatment differences between the United States and Europe; however, inclusion criteria in LocoMMotion were uniform across countries in terms of number of prior LOT and triple-class exposure, minimizing the impact of differences in frontline treatment. Additionally, LocoMMotion was a single-arm, open-label, prospective study with no comparator group. In real-world studies, some baseline information and laboratory assessments required per IMWG criteria might be missing. However, a framework was implemented by RRC to avoid underestimation of response and to ensure unified evaluation across all study participants.

One limitation of the study may be possible selection bias for enrolling patients with less advanced disease or poorer overall fitness into this observational study versus into interventional trials. Unadjusted comparisons between the LocoMMotion population and populations in interventional trials like CARTITUDE-1 and MajesTEC-1 have shown some imbalances in patient characteristics, such as in refractory status, time to progression on prior LOT, and duration of prior LOT. Hence, indirect treatment comparisons using the LocoMMotion study population as an external control arm were adjusted for baseline differences, and major findings were published [15, 16].

Sample sizes of some subgroups were smaller, and the non-randomized design of the study may have led to unbalanced subgroups with respect to other characteristics. Also, PFS/OS analysis by achievement of VGPR is a post-baseline measurement that complicates interpretation because response is partly a consequence of PFS/OS.

Finally, the observational nature of LocoMMotion likely resulted in underreporting of TEAEs, and the reporting period for AEs was limited from the start of index LOT treatment to the end of index LOT. Therefore, safety of RWCP therapies should be interpreted with caution given that the median time on RWCP therapy was only 4 months.

In conclusion, the final (2-year) analysis of the LocoMMotion study confirms a lack of clear standard of care treatment and poor outcomes for triple-class exposed patients with RRMM. Hopefully, the emergence of novel therapies, including chimeric antigen receptor T-cell therapies and bispecific T-cell redirecting therapies, will improve outcomes for heavily pretreated patients with MM. Therapeutics in these treatment classes were approved for use in the United States [17–19] and in Europe [20–22] after the enrollment period for LocoMMotion. Data from the LocoMMotion study are a valuable benchmark for comparison with newly approved and emerging therapies [15]. The prospective design, comprehensive data collection, and the completeness of the baseline characteristics data allow for comparative analyses with other single-arm trials in this patient population.

## Supplementary information

Table S1: Ethics committees/Institutional Review Boards in LocoMMotion

Table S2: Regimens received by ≥3 patient in any given LOT.

Table S3: TEAEs reported in ≥5% of patients in index LOT.

Figure S1: Forest plot of subgroup analyses of progression-free survival and overall survival by RRC.

## Data Availability

Although these data are not currently publicly available for sharing, requests for sharing can be sent to the Corresponding Author and will be evaluated on an individual basis.
