# Supplementary material for: LocoMMotion: a study of real-life current standards of care in triple-class exposed patients with relapsed/refractory multiple myeloma – 2-year follow-up (final analysis)
Source: Leukemia. 2024 Sep 25;38(12):2554–60. doi: 10.1038/s41375-024-02404-6 (PMC11588650; doi:10.1038/s41375-024-02404-6)
Supplement: Supplementary file 1 — Table S1: Ethics committees/Institutional Review Boards in LocoMMotion [file 41375_2024_2404_MOESM1_ESM.pdf]

Table S1: Ethics committees/Institutional Review Boards in LocoMMotion

Country	Ethics Committee/ Institutional Review Board address	Approval date	Ref #
US	The James/ A054 Starling Loving Hall<br>320 W. 10th Ave, Columbus, OH 43210	15 Nov 2019	OSU-19288
US	Columbia Research<br>615 West 131st St. 3rd floor, New York, NY 10027	21 Nov 2019	IRB-AAAS7369 or 7571412
US	Washington University in St. Louis<br>660 South Euclid Ave. Campus Box 8089, St. Louis MO 63110	19 Dec 2019	201912009
US	Sterling IRB<br>6300 Powers Ferry Road Suite 600-351, Atlanta GA 30339	10 Oct 2019	IRB ID 7513-PDEisenberg
US	Sterling IRB<br>6300 Powers Ferry Road Suite 600-351, Atlanta GA 30339	29 Oct 2019	IRB ID 7513-Amazumder
US	Sterling IRB<br>6300 Powers Ferry Road Suite 600-351, Atlanta GA 30339	19 Nov 2019	IRB ID 7513-KSJahangir
US	Sterling IRB<br>6300 Powers Ferry Road Suite 600-351, Atlanta GA 30339	21 Oct 2019	IRB ID 7513-MEBednar
US	Sterling IRB<br>6300 Powers Ferry Road Suite 600-351, Atlanta GA 30339	13 Nov 2019	IRB ID 7513-Cchen

US	Sterling IRB<br>6300 Powers Ferry Road Suite 600-351, Atlanta GA 30339	3 Dec 2019	IRB ID 7513-DHuang
US	Sterling IRB<br>6300 Powers Ferry Road Suite 600-351, Atlanta GA 30339	30 Sep 2019	IRB ID 7513-Zmalik
US	Sterling IRB<br>6300 Powers Ferry Road Suite 600-351, Atlanta GA 30339	30 Oct 2019	IRB ID 7513-Lshunyakov
US	Sterling IRB<br>6300 Powers Ferry Road Suite 600-351, Atlanta GA 30339	21 Jan 2020	IRB ID 7513-Hterebolo
US	St. Francis Hospital The Heart Center	5 Dec 2019	IRB #19-34
US	Sterling IRB<br>6300 Powers Ferry Road Suite 600-351, Atlanta GA 30339	4 Dec 2019	IRB ID 7513-RRCaradonna
US	Sterling IRB<br>6300 Powers Ferry Road Suite 600-351, Atlanta GA 30339	5 Nov 2019	IRB ID 7513-Mbajaj
Germany	EC Universiaet Heidelberg, Ethik-Kommission I der Medizinischen Fakultät Heidelberg, Alte Glockengiesserei 11/1, 69115 Heidelberg	16 Aug 2019	S-549/2019
Germany	Tuebingen Ethik-Kommission an der Medizinischen Fakultät der Eberhard-Karls-Universität und am Universitätsklinikum Tübingen<br>Gartenstraße 47, 72074 Tübingen	16 Sep 2019	548/2019BO2

Germany	EC medizinische Hochschule Hannover Ethik-Kommission d. Medizinischen Hochschule Hannover<br>Carl-Neuberger-Str. 1, 3623 Hannover	20 Sep 2019	8672_B0_K_2019
Germany	EC Aertzekammer Hamburg Ethikkommission der Ärztekammer Hamburg; Körperschaft des öffentlichen Rechts<br>Weidestr. 122b, 22083 Hamburg	21 Oct 2019	PV7092
Germany	EC Universitaet Wuerzburg<br>Josef-Schneider-Str. 4, Bau C15, 97080 Wuerzburg	17 Jan 2020	172/19-me
Germany	EC Universiaet Koeln Geschäftsstelle der ethik-kommission der medizinischen fakultät der universität zu köln<br>Kerpenerstr 62, 50937 Köln	18 Feb 2020	19-1545_1
Spain	Comité Coordinador de Ética de la Investigación Clínica de Andalucía<br>Avda. de la Innovación, s/n. Edificio Arena 1, 41080 Sevilla	2 Oct 2019	N/A
Spain	Comité Coordinador de Ética de la Investigación Clínica de Andalucía<br>Avda. de la Innovación, s/n. Edificio Arena 1, 41080 Sevilla	7 Oct 2019	N/A
Spain	Comité Coordinador de Ética de la Investigación Clínica de Andalucía<br>Avda. de la Innovación, s/n. Edificio Arena 1, 41080 Sevilla	7 Oct 2029	N/A
Spain	Comité de Ética de la Investigación de Cadiz<br>Avda. Ana de Viya, 21, 11009 Cadiz	23 Jul 2020	N/A

Spain	Comité Coordinador de Ética de la Investigación Clínica de Andalucía<br>Avda. de la Innovación, s/n. Edificio Arena 1, 41080 Sevilla	30 Jul 2020	N/A
UK	Tayside medical science centre<br>Residency block level 3, George Pirie way, Ninewells hospital and medical school, Dundee, DD1 9SY	29 Jul 2019	DL/19/ES/0088
Italy	Comitato Etico<br>Area 3 ASL Lecce, Via Miglietta 5,73100	19 Sep 2019	CE150036
Italy	Comitato Etico Regione Liguria<br>Largo Rosanna Benzi 10, 16132 Genova	24 Jan 2020	CE150193
Italy	Comitato etico dell'IRCCS casa sollievo della sofferenza di s. giovanni rotondo<br>Viale Cappuccini, 71013 SGR	17 Jul 2019	CE150037
Italy	Comitato Etico Indipendente Azienda Ospedaliera Universitaria Policlinico S. Orsola-Malpighi di Bologna<br>Via Albertoni 15, 40138 Bologna	3 Oct 2019	CE150192
Italy	Comitato Etico della Fondazione Policlinico Universitario Agostino Gemelli IRCCS Università Cattolica del Sacro Cuore<br>Largo Agostino Gemelli 8, 00168 Roma	22 Jul 2019	CE150057
Italy	Comitato Etico Catania 1 c/o AOU Policlinico Vittorio Emanuele<br>Via Santa Sofia 78, Catania	30 Oct 2019	CE150101

Italy	Comitato Etico "La Sapienza" Azienda Policlinico Umberto I	24 Sep 2019	CE150031
	Via Del Policlinico 155, 00161 Roma		
Italy	Comitato Etico interaziendale aou citta' della salute e della scienza di Torino	25 Oct 2019	CE150115
	Corso Bramante 88/90, 10126 Torino		
Italy	CESC della Provincia di Padova	25 Jun 2020	CE150028
	Via Giustininani 1, 35128 Padova		
Italy	Comitato Etico Palermo 2 IRB-EC	14 Oct 2019	CE150125
	Viale Strasburgo 233, 90136 Palermo		
Italy	Comitato etico referente per l'area di Pavia	5 Sep 2019	CE150183
	Viale Golgi 19, 27100 Pavia		
Italy	Comitato Etico Unico Regionale per la Basilicata IRB-EC	16 Oct 2019	CE150051
	Via Potito Petrone 1, 85100 Potenza		
Italy	Comitato Etico Interregionale c/o A.O.U. Policlinico Consorziale	8 Oct 2019	CE150162
	Piazza Giulio Cesare 11, 70124 Bari		
Italy	Comitato Etico IRCCS Istituto Tumori "Giovanni Paolo II"	23 Apr 2020	CE150168
	Viale orazio flacco 65, 70124 Bari		
Italy	Comitato Etico della Romagna CEROM	8 May 2020	CE150190
	Via Piero Maroncelli 40, 47014 Meldola		

Italy	Comitato Etico Regione Toscana - Area Vasta Centro c/o Azienda Ospedaliera Universitaria Careggi<br>Largo Brambilla 3, 50134 Firenze	11 May 2020	CE150071
Belgium	Etische Commissie, Onderzoek UZ/KU Leuven<br>Herestraat 49, 3000 Leuven	13 Sep 2019	S62984
The Netherlands	METC van de stichting BEBO<br>Dr. Nassaulaan 10, 9401 HK Assen	16 Jul 2019	19.084/IH
Poland	Komisja Bioetyczna przy, Uniwersytecie Medycznym im. Karola Marcinkowskiego w Poznaniu, Collegium Maius<br>Dział Badań Naukowych; ul. Bukowska 70, 60-812 Poznań	07 Nov 2019	991/19
France	CPP EST III, Hopital de Brabois<br>Rue du Morvan, 54511 Vandoeuvre-les-Nancy Cedex	5 Sep 2019	Numéro ID RCB:<br>2019-A01716-51
Russia	Independent Interdisciplinary Committee on Ethics Expertise of Clinical Trials,<br>51 Leningradskiy prospect, 125468 Moscow	4 Oct 2019	15
