# Supplementary material for: LocoMMotion: a study of real-life current standards of care in triple-class exposed patients with relapsed/refractory multiple myeloma – 2-year follow-up (final analysis)
Source: Leukemia. 2024 Sep 25;38(12):2554–60. doi: 10.1038/s41375-024-02404-6 (PMC11588650; doi:10.1038/s41375-024-02404-6)
Supplement: Supplementary file 2 — Table S2: Regimens received by ≥3 patient in any given LOT. [file 41375_2024_2404_MOESM2_ESM.pdf]

Table S2: Regimens received by ≥3 patient in any given LOT.[a]

Regimen, *n* (%)	Index LOT (*N* = 248)	Subsequent LOT 1st (*n* = 152)	2nd (*n* = 74)	3rd (*n* = 42)	≥4th (*n* = 17)
Cyclophosphamide-Dexamethasone-Pomalidomide	35 (14.1)	10 (6.6)	1 (1.4)	1 (2.4)	0 (0)
Carfilzomib-Dexamethasone	35 (14.1)	5 (3.3)	4 (5.4)	0 (0)	0 (0)
Dexamethasone-Pomalidomide	29 (11.7)	4 (2.6)	2 (2.7)	0 (0)	0 (0)
Dexamethasone-Ixazomib-Lenalidomide	14 (5.6)	4 (2.6)	1 (1.4)	1 (2.4)	1 (5.9)
Bortezomib-Dexamethasone-Panobinostat	11 (4.4)	5 (3.3)	0 (0)	0 (0)	0 (0)
Carfilzomib-Cyclophosphamide-Dexamethasone	7 (2.8)	5 (3.3)	4 (5.4)	0 (0)	0 (0)
Bendamustine-Bortezomib-Dexamethasone	7 (2.8)	4 (2.6)	1 (1.4)	0 (0)	0 (0)
Dexamethasone-Elotuzumab-Pomalidomide	6 (2.4)	4 (2.6)	0 (0)	0 (0)	1 (5.9)
Dexamethasone-Lenalidomide	6 (2.4)	1 (0.7)	1 (1.4)	0 (0)	0 (0)
Bortezomib-Dexamethasone-Doxorubicin	5 (2)	2 (1.3)	1 (1.4)	0 (0)	0 (0)
Carfilzomib-Dexamethasone-Pomalidomide	5 (2)	2 (1.3)	1 (1.4)	0 (0)	0 (0)
Carfilzomib-Dexamethasone-Lenalidomide	5 (2)	0 (0)	1 (1.4)	0 (0)	0 (0)
Belantamab Mafodotin	4 (1.6)	16 (10.5)	14 (18.9)	8 (19)	3 (17.6)
Cyclophosphamide-Dexamethasone	4 (1.6)	2 (1.3)	0 (0)	1 (2.4)	0 (0)
Melphalan	4 (1.6)	2 (1.3)	0 (0)	0 (0)	0 (0)
Bendamustine-Prednisone	4 (1.6)	1 (0.7)	1 (1.4)	0 (0)	0 (0)
Dexamethasone	3 (1.2)	3 (2)	0 (0)	0 (0)	1 (5.9)
Bortezomib-Dexamethasone-Venetoclax	3 (1.2)	2 (1.3)	0 (0)	0 (0)	0 (0)
Carfilzomib-Daratumumab-Dexamethasone	3 (1.2)	1 (0.7)	1 (1.4)	2 (4.8)	0 (0)
Dexamethasone-Melphalan	3 (1.2)	1 (0.7)	1 (1.4)	0 (0)	0 (0)
Bortezomib-Daratumumab-Dexamethasone	3 (1.2)	1 (0.7)	0 (0)	0 (0)	1 (5.9)
Dexamethasone-Ixazomib	3 (1.2)	1 (0.7)	0 (0)	0 (0)	0 (0)
Dexamethasone-Elotuzumab-Lenalidomide	3 (1.2)	1 (0.7)	0 (0)	0 (0)	0 (0)
Cisplatin-Cyclophosphamide-Dexamethasone-Doxorubicin-Etoposide	3 (1.2)	1 (0.7)	0 (0)	1 (2.4)	0 (0)
Bortezomib-Cyclophosphamide-Dexamethasone	3 (1.2)	0 (0)	1 (1.4)	1 (2.4)	1 (5.9)
Bortezomib-Dexamethasone-Lenalidomide	3 (1.2)	0 (0)	0 (0)	0 (0)	1 (5.9)
Daratumumab-Dexamethasone-Pomalidomide	3 (1.2)	0 (0)	0 (0)	0 (0)	1 (5.9)
Carfilzomib	3 (1.2)	0 (0)	0 (0)	0 (0)	0 (0)
Dexamethasone-Ixazomib-Pomalidomide	3 (1.2)	0 (0)	0 (0)	0 (0)	0 (0)
Carfilzomib-Cisplatin-Cyclophosphamide-Daratumumab-Etoposide	3 (1.2)	0 (0)	0 (0)	0 (0)	0 (0)
Bendamustine	2 (0.8)	3 (2)	3 (4.1)	1 (2.4)	0 (0)

Cyclophosphamide-Dexamethasone-Thalidomide	1 (0.4)	5 (3.3)	3 (4.1)	1 (2.4)	0 (0)
Bortezomib-Dexamethasone-Selinexor	0 (0)	5 (3.3)	1 (1.4)	0 (0)	0 (0)
Teclistamab	0 (0)	4 (2.6)	2 (2.7)	0 (0)	1 (5.9)
Dexamethasone-Selinexor	0 (0)	3 (2)	1 (1.4)	1 (2.4)	1 (5.9)
Dexamethasone-Isatuximab-Pomalidomide	0 (0)	2 (1.3)	1 (1.4)	5 (11.9)	1 (5.9)

CAR chimeric antigen receptor, *LOT* line of therapy.
[a]Patients can be counted in ≥1 regimen.
