# Supplementary material for: LocoMMotion: a study of real-life current standards of care in triple-class exposed patients with relapsed/refractory multiple myeloma – 2-year follow-up (final analysis)
Source: Leukemia. 2024 Sep 25;38(12):2554–60. doi: 10.1038/s41375-024-02404-6 (PMC11588650; doi:10.1038/s41375-024-02404-6)
Supplement: Supplementary file 3 — Table S3: TEAEs reported in ≥5% of patients in index LOT. [file 41375_2024_2404_MOESM3_ESM.pdf]

Table S3: TEAEs reported in ≥5% of patients in index LOT.[a]

TEAE	Total (*N* = 248)	
	**Any grade, *n* (%)**	**Grade 3/4, *n* (%)**
Hematologic TEAE	124 (50.0)	101 (40.7)
Thrombocytopenia	65 (26.2)	48 (19.4)
Anemia	64 (25.8)	27 (10.9)
Neutropenia	50 (20.2)	43 (17.3)
Leukopenia	22 (8.9)	15 (6.0)
Lymphopenia	21 (8.5)	19 (7.7)
Non-hematologic TEAEs		
Infections and infestations	82 (33.1)	20 (8.1)
Nervous system disorders	60 (24.2)	8 (3.2)
Peripheral sensory neuropathy	15 (6.0)	2 (0.8)
General disorders and administration site conditions	101 (40.7)	16 (6.5)
Pyrexia	33 (13.3)	6 (2.4)
Fatigue	31 (12.5)	2 (0.8)
Asthenia	24 (9.7)	3 (1.2)
Peripheral edema	24 (9.7)	1 (0.4)
Gastrointestinal disorders	83 (33.5)	9 (3.6)
Diarrhea	40 (16.1)	2 (0.8)
Nausea	25 (10.1)	3 (1.2)
Constipation	14 (5.6)	0
Vomiting	14 (5.6)	2 (0.8)
Musculoskeletal and connective tissue disorders	67 (27.0)	15 (6.0)
Back pain	26 (10.5)	4 (1.6)
Arthralgia	20 (8.1)	3 (1.2)
Bone pain	13 (5.2)	2 (0.8)
Respiratory, thoracic, and mediastinal disorders	55 (22.2)	14 (5.6)
Dyspnea	29 (11.7)	5 (2.0)
Metabolism and nutrition disorders	37 (14.9)	9 (3.6)
Injury, poisoning, and procedural complications	30 (12.1)	10 (4.0)
Psychiatric disorders	26 (10.5)	3 (1.2)
Renal and urinary disorders	25 (10.1)	14 (5.6)
Investigations	24 (9.7)	7 (2.8)
Skin and subcutaneous tissue disorders	23 (9.3)	1 (0.4)
Vascular disorders	22 (8.9)	7 (2.8)
Cardiac disorders	18 (7.3)	8 (3.2)
Eye disorders	18 (7.3)	4 (1.6)

LOT line of therapy, *TEAE* treatment-emergent adverse event.
