# Supplementary material for: LocoMMotion: a study of real-life current standards of care in triple-class exposed patients with relapsed/refractory multiple myeloma – 2-year follow-up (final analysis)
Source: Leukemia. 2024 Sep 25;38(12):2554–60. doi: 10.1038/s41375-024-02404-6 (PMC11588650; doi:10.1038/s41375-024-02404-6)
Supplement: Supplementary file 4 — Figure S1: Forest plot of subgroup analyses of progression-free survival and overall survival by RRC. [file 41375_2024_2404_MOESM4_ESM.pdf]

Fig. S1: Forest plot of subgroup analyses of progression-free survival and overall survival by RRC

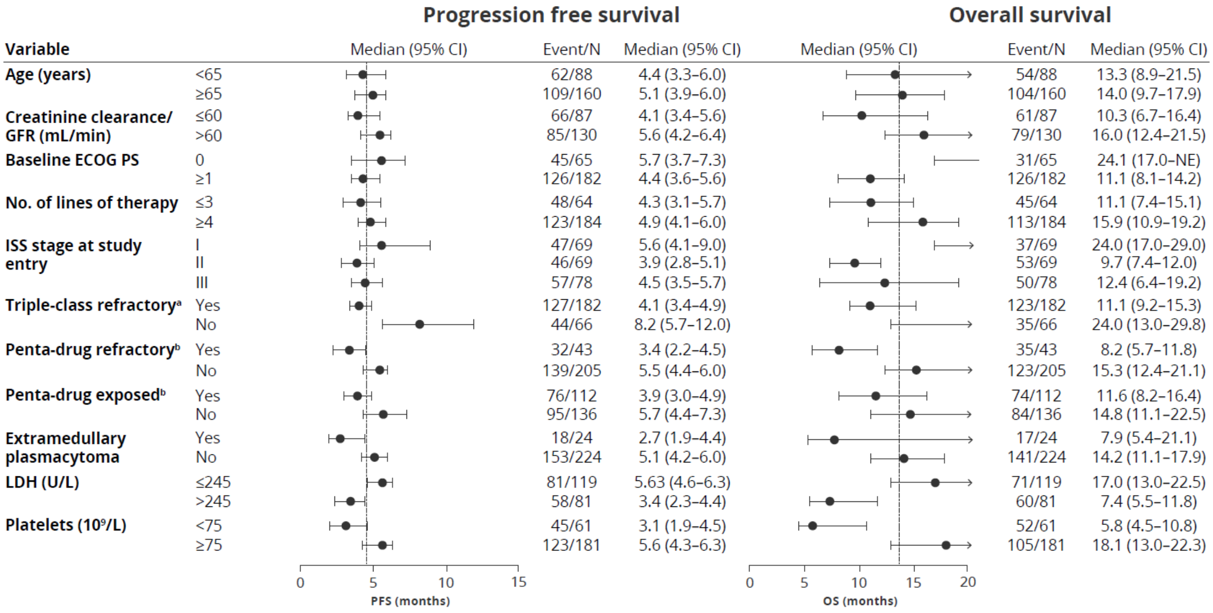

ECOG Eastern Cooperative Oncology Group, *GFR* glomerular filtration rate, *ISS* International staging system, *LDH* lactate dehydrogenase, *PS* performance status, *RRC* response review committee. [a]Triple-class exposed/refractory is defined as exposed/refractory to a proteasome inhibitor (PI), an immunomodulatory drug (IMiD), and an anti-CD38 antibody. [b]Penta-drug exposed/refractory is defined as exposed/refractory to at least 2 PIs, 2 IMiD, and 1 anti-CD38 antibody (includes triple-class exposed/refractory).
